# Isolation and characterization of phage display-derived scFv antibodies against human parechovirus 1 VP0 protein

**DOI:** 10.1038/s41598-022-17678-y

**Published:** 2022-08-04

**Authors:** Eero Hietanen, Lav Tripathi, Eeva-Christine Brockmann, Pirjo Merilahti, Urpo Lamminmäki, Petri Susi

**Affiliations:** 1 Institute of Biomedicine, University of Turku, Turku, Finland; 2 Department of Biotechnology, University of Turku, Turku, Finland; 3 Present Address: Biovian Ltd, Turku, Finland

**Keywords:** Virology, Antibody fragment therapy

## Abstract

Human parechoviruses (PeVs) are common viruses that are associated with a variety of diseases from mild gastrointestinal and respiratory symptoms to severe central nervous system infections. Until now there has not been antibodies for visualizing parechovirus infection. We used *E. coli* recombinant PeV-A1-VP0 protein as a target in phage display single chain variable fragment (scFv) antibody library panning. Three rounds of panning allowed identification and isolation of several candidate scFv clones, which tested positive in enzyme-linked immunosorbent assay (ELISA) against VP0. Three scFv clones (scFv-55, -59 and -71) with different CDR-3 sequences were further purified and tested in ELISA, Western blot and immunofluorescence microscopy (IFA) against a set of PeV-A1 isolates and a few isolates representing PeV types 2–6. In IFA, all three scFv binders recognized twenty PeV-A1 isolates. ScFv-55 and -71 also recognized clinical representatives of PeV types 1–6 both in IFA and in capture ELISA, while scFv-59 only recognized PeV-A1, -A2 and -A6. PeV-A1-VP0 (Harris strain) sequence was used to generate a peptide library, which allowed identification of a putative unique conformational antibody epitope with fully conserved flanking regions and a more variable core VVTYDSKL, shared between the scFv antibodies. Sequencing of the VP0 region of virus samples and sequence comparisons against parechoviral sequences in GenBank revealed 107 PeV-A1, -A3, -A8, -A17, -A (untyped) sequences with this exact epitope core sequence, which was most dominant among PeV-A1 isolates. These data suggest the first-time isolation of broad range phage display antibodies against human parechoviruses that may be used in diagnostic antibody development.

## Introduction

Human parechoviruses (PeVs) are common viruses that are associated with a variety of diseases from mild gastrointestinal or respiratory symptoms to severe central nervous system infections^1^. They belong to family *Picornaviridae*, genus *Parechovirus* in *Parechovirus A* (*PeV-A*) species. Currently, nineteen PeV-A types have been identified and named (www.picornaviridae.com). PeVs encode three structural viral proteins (VP), VP0, VP1 and VP3 from their single-stranded, positive-sense RNA genome^2^. VP0 is a fusion protein between VP4 and VP2 proteins, which are commonly found in other species of human picornaviruses. PeVs mainly infect neonates and young children, and are the second most important cause of viral sepsis-like illness and meningitis in infants^3^. Clinical diagnosis of human parechoviruses is by using specific RT-qPCR, which targets the 5′-terminal untranslated region of the viral RNA genome^4,5^. More recently parechoviruses have been dualplexed with enteroviruses in RT-qPCR due to their prevalence in CNS disease^6,7^. Parechovirus is also included in some multiplex assays for respiratory pathogens. For antigen detection there are only a few antibodies available, but they are not in clinical use^8,9^.

Recombinant antibody phage library technology is an efficient method to generate antibodies without immunization^10^. Binders with desired specificity can be enriched from a vast naïve recombinant antibody repertoire through the panning process comprising iterative rounds of affinity capture with the target antigen and amplification of the captured binders. In comparison to immunization methods, the amount of antigen needed for the panning is relatively small and different forms of the antigens can easily be used at the different selection rounds, which, in turn, can help to direct the antibodies towards particular regions or epitopes in the target. Moreover, there are not many restrictions regarding the type of target antigen. For example, conserved and toxic molecules, protein–ligand-complexes^11^ and intact supramolecular structures such as HDL-particles^12^ can be used as targets. Many features of phage display antibody library technology can be useful considering diagnostic and therapeutic antibody development against viral pathogens.

Capsid proteins have traditionally been used in development of diagnostic measures against viral pathogens. However, there are only a few papers which describe diagnostic antibody development against human parechoviruses. Diagnostic potential of parechoviral polyclonal antibodies has only been evaluated in a single paper against PeV-A1^13^ while parechoviral VP0 and VP1 recombinant proteins have been used as an antigen in some serological studies^14,15^. We have recently used recombinant PeV-A1-VP0 protein (from Harris strain) to develop polyclonal antiserum and a single monoclonal antibody that recognize PeV types 1 to 6^8^. In this study, we used this protein and intact PeV-A1 virus (Harris strain) as targets in phage display antibody panning to isolate virus-specific human scFv antibody fragments. The resulting antibodies recognized several cultivable parechovirus types (types 1 to 6) including clinical specimens. We also identified a putative conformational antibody epitope by using peptide scanning and further analysed its prevalence in all GenBank parechovirus genome sequences.

## Results

### Library screening and sequence analysis of PeV1-A1-VP0 binders

**Figure 1:**
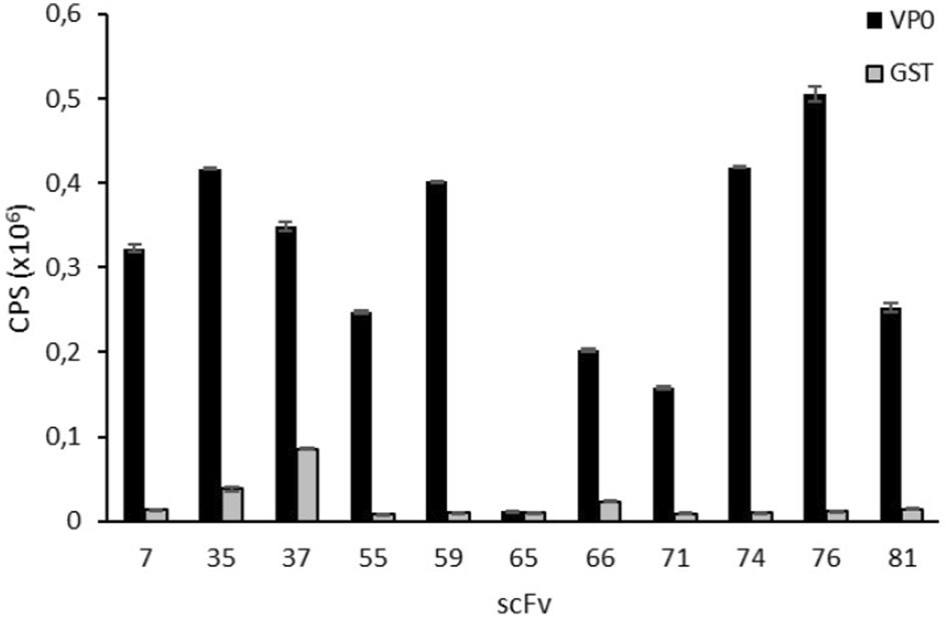
Time-resolved fluoroimmunoassay of selected scFv clones. Based on CDR-H3 sequences, a total of 11 clones were selected for secondary screening against VP0-GST and GST in a TRF-assay using europium chelate. In the assay, His-tag purified scFv clones expressed as a fusion to bacterial alkaline phosphatase, His-tag and FLAG tag were bound to biotinylated VP0-GST and biotinylated GST on 96 well streptavidin microtiter plate. Bound scFv-AP was detected with polyclonal Eu-labelled anti-alkaline phosphatase antibody and time-resolved fluorescence from europium was measured with Victor^3^ multilabel counter. The Y-axis represents the europium counts per second. The X-axis represents the different clones screened based on different CDR-H3 sequence.

Recently, we demonstrated that monoclonal antibody produced against PeV-A1-VP0 protein recognized six PeV types suggestive of shared epitope within these types^8^. In this study, we used the same antigen to isolate broad range detector antibodies from synthetic scFv phage display antibody library. Three rounds of panning against PeV-A1-VP0 were performed using scFvP and scFvM libraries to enrich PeV-A1-VP0-specific binders. These two synthetic libraries differ in terms of their binding site designs and hence there are differences in their specificity profiles; scFvM shows capacity to produce binders against both low molecular weight as well as macromolecular antigens whereas scFvP is more restricted to targeting macromolecules^16^. To ensure that the obtained scFv binders bind to the native virus, the third round of panning was performed using purified PeV-A1 (Harris strain) particles in parallel to PeV-A1-VP0. The scFv genes from enriched libraries were cloned into pLK06FT to generate scFv-AP (bacterial alkaline phosphatase) His6-FLAG tag fusion proteins for primary ELISA screening. In the primary screening, 9/96 and 37/96 of the clones isolated after 2 and 3 rounds of PeV-1-VP0 panning, and 44/96 clones isolated after the third round of PeV-A1 virus panning, respectively, showed strong binding (A_405_ > 1.5; background signal < 0.1) to PeV-A1-VP0 (data not shown). A total of 84 clones showing elevated signals in primary ELISA screen were Sanger-sequenced and 68 clones were analysed further. Among these 68 sequenced clones, 18 unique sequences with 17 different heavy chain complementarity determining region 3 (CDR-H3) were identified. Three of the clones originated from the scFvP and 15 from the scFvM library as deduced from the library specific sequence features. Six clones were exclusively obtained from a panning round with PeV-A1 virus particles and seven from VP0 recombinant protein panning round, while the remaining five clones were found in both PeV-A1 and VP0 pannings. In all, eleven (11) clones were further selected for IMAC (immobilized metal affinity chromatography) purification, all of which were found in pannings that included a round on the virus particles. IMAC-purified scFv antibodies were used in a time-resolved fluorescence (TRF) assay against VP0-GST and GST (Fig. 1), and further tested in immunofluorescence assay (IFA) against PeV-A1 (data not shown). Based on the results, combined with observed better growth characteristics of the selected clones, three binders (named as scFv-55, scFv-59 and scFv-71) derived from the library scFvM were selected for further assays. The sequences of CDR3 regions are shown in the Table 1. Please see Supplementary Figure S5 for more detailed data regarding the single clone sequences.

**Table 1:** Variable heavy and light chain single clone sequences of scFv-55, scFv-59, and scFv-71. Positions with differences present are marked bold.

Name	Light chain (human KV3-20)		Heavy chain (human HV3-23)		
	CDR-L1	CDR-L3	CDR-H1	CDR-H2	CDR-H3
IMGT No.	38–40	105–117	36–40	55–66	105–117
scFv-55	YL**H**	**L**Q**DNYD**P**P**T	SY**G**M**D**	**R**ITPSGG**STY**	**ASGAY**
scFv-59	YL**N**	**Q**Q**TYYI**P**L**T	SY**A**M**S**	**S**ITPSGG**EHG**	**TGWAY**
scFv-71	YL**H**	**L**Q**DNSA**P**L**T	SY**G**M**D**	**R**ITPSGG**STY**	**ASGYI**

### Binding of the scFv antibodies to PeV-A1 virus and PeV-A1-VP0-GST recombinant protein

**Figure 2:**
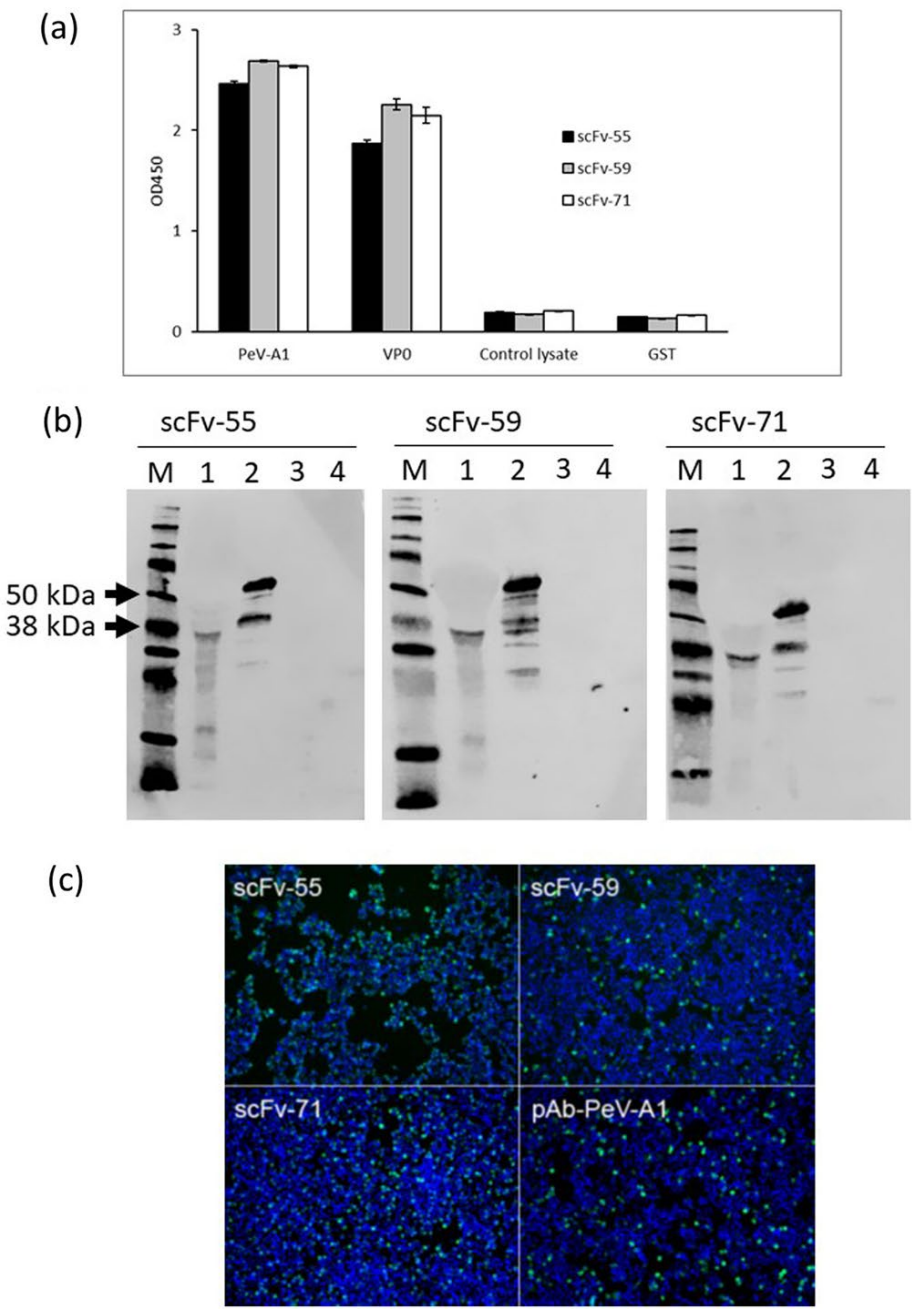
Determination of binding properties of scFv antibodies by sandwich ELISA, Western blot and IFA. (**a**) Sandwich ELISA assay of scFv antibodies was performed against PeV-A1, VP0, cell control lysate and GST. 96-well plate was coated with scFv-AP antibodies (500 ng/well) followed by addition of PeV-A1 virus, VP0 protein, control lysate and GST protein. Rabbit anti-PeV antiserum (pAb-PeV-A1) was used as detector antibody. HRP-conjugated anti-rabbit secondary antibody was added followed by the substrate. The reaction was stopped with H_2_SO_4_ and OD_450_ was measured with Victor^3^ multilabel counter. (**b**) Detection of PeV-A1-VP0 by Western blot. M: marker; lane 1: PeV-A1 (Harris strain); lane 2: VP0-GST; lane 3: Cell lysate; lane 4: GST. The molecular weight of PeV-A1-VP0 protein is approximately 37 kDa and that of VP0-GST fusion protein about 64 kDa. (**c**) Detection of PeV-A1 with scFv and rabbit anti-PeV-A1. The HT-29 cells were infected with PeV-A1 (Harris strain), fixed, permeabilized and stained with scFv antibodies and polyclonal anti-PeV-A1 antiserum as a control. Virus is shown as green, and nuclei stained with DAPI in blue. Stained cells were visualized using EVOS FL Auto imaging microscope (10 × magnification).

The specific activities of three purified scFv antibodies were first analysed against PeV-A1 (Harris strain) and PeV-A1-VP0-GST protein by sandwich ELISA, Western blot and IFA. Non-infected cell lysate was used as a control against virus. Purified GST protein was used as a control against scFv binders because VP0-GST had been used as a target in phage display antibody library panning. In sandwich ELISA, 96-well plate was coated with scFv antibodies followed by adding of PeV-A1 (Harris strain), VP0 protein, cell lysate or GST protein. As shown in Fig. 2a, PeV-A1 and VP0 were efficiently recognized by all three scFv antibodies, while binding to cell lysate or GST was not observed. In Western blot (Fig. 2b), all three scFv antibodies recognized a protein of approximately 37 kDa, which corresponds to the molecular weight (MW) of native PeV-A1-VP0 (lane 1), and also VP0-GST fusion protein with the MW of 64 kDa (lane 2). ScFv antibodies also bound to protein fragments with other sizes, which may be indicative of degradation of VP0-GST fusion protein. Instead, scFv antibodies did not recognize purified GST protein, and there was no binding to non-infected cell lysate proteins. Furthermore, scFv antibodies were tested in IFA (Fig. 2c). HT-29 cells were grown on 96-well plate and infected with PeV-A1 (Harris strain). After infection, the cells were fixed, permeabilized and stained with scFv antibodies followed by imaging with EVOS FL Auto microscope. ScFv antibodies recognized PeV-A1 (Harris strain) similarly to the anti-PeV-A1 polyclonal antiserum (pAb-PeV-A1)^8^ used as a positive control.

### Detection of PeV-A1 isolates by scFv antibodies in immunofluorescence assay

**Figure 3:**
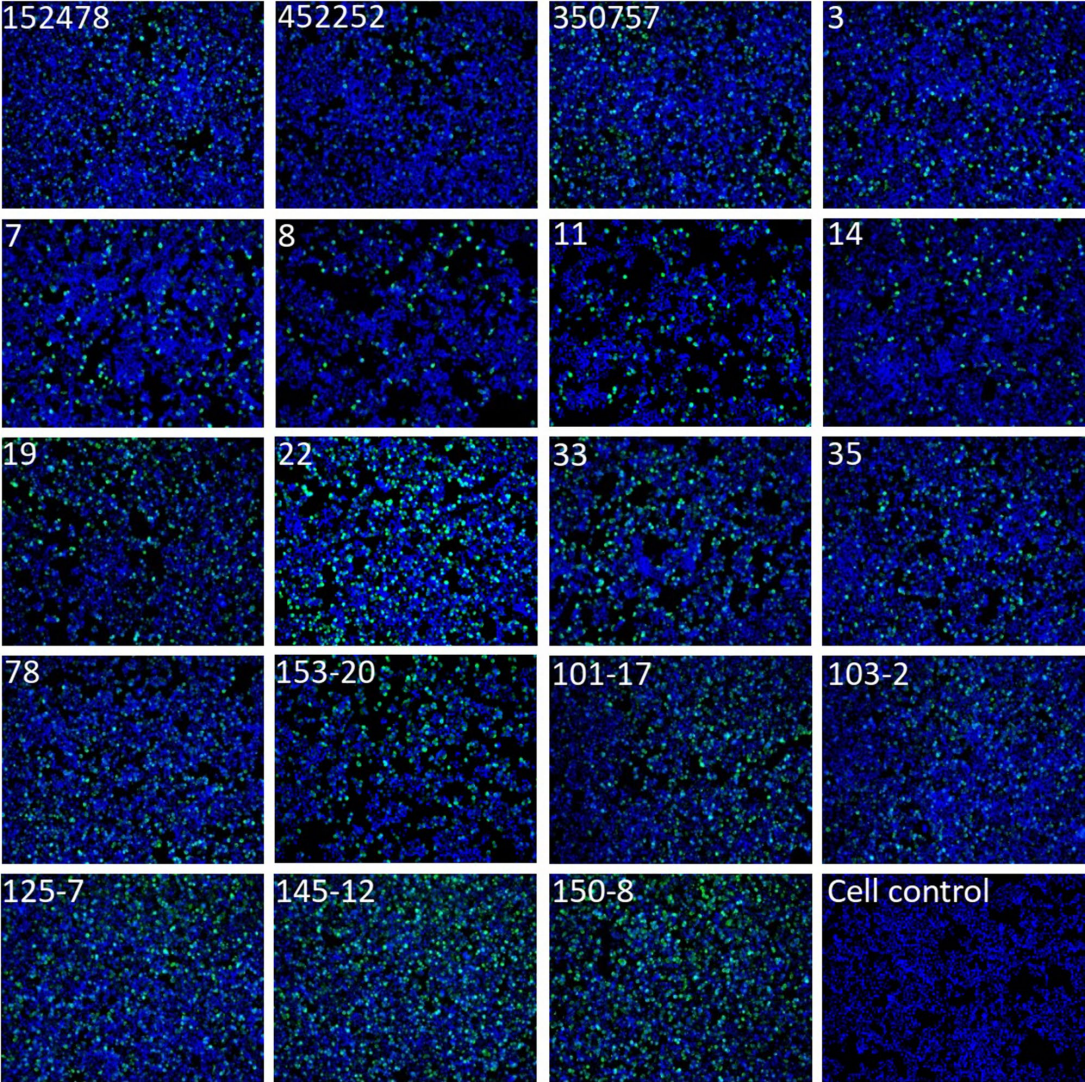
Detection of PeV-A1-infected HT-29 cells with scFv-71. HT-29 cells were mock-inoculated or inoculated with 19 clinical PeV-A1 isolates followed by staining with scFv-71 antibody and rabbit anti-FLAG antibody followed by anti-rabbit Alexa Fluor 488 secondary antibody. Nuclei were stained with DAPI. Stained cells were visualized using EVOS FL Auto imaging microscope (10 × magnification).

To further analyse broad range specificity of the three scFv antibodies, they were tested against 19 clinical PeV-A1 isolates from different sources in immunofluorescence assay (IFA). HT-29 cells were grown on 96-well plate and infected with PeV-A1 isolates with pre-tested virus amount equivalent to infection efficiency of approximately 20–40% FFUs. After six hours of infection, the cells were fixed, permeabilized and stained with scFv antibodies followed by incubation with anti-FLAG and Alexa Fluor 488 secondary antibodies. Nuclei were stained with DAPI. Plates were imaged with EVOS FL Auto microscope. While Fig. 3 shows the IFA results for scFv-71, all three scFv antibodies positively stained all 19 PeV-A1 isolates (Table 2). The data indicate that the binding site is well-conserved between the PeV-A1 isolates and that the binding is very specific (no background was detected).

**Table 2:** Summary of the data from immunofluorescence microscopy assay.

Type	Isolate	scFv-55	scFv-59	scFv-71
PeV-A1	Harris	+	+	+
PeV-A1	152478	+	+	+
PeV-A1	452252	+	+	+
PeV-A1	350757	+	+	+
PeV-A1	3	+	+	+
PeV-A1	7	+	+	+
PeV-A1	8	+	+	+
PeV-A1	11	+	+	+
PeV-A1	14	+	+	+
PeV-A1	19	+	+	+
PeV-A1	22	+	+	+
PeV-A1	33	+	+	+
PeV-A1	35	+	+	+
PeV-A1	78	+	+	+
PeV-A1	153-20	+	+	+
PeV-A1	101-17	+	+	+
PeV-A1	103-2	+	+	+
PeV-A1	125-7	+	+	+
PeV-A1	145-12	+	+	+
PeV-A1	150-8	+	+	+
PeV-A2	Williamson	+	+	+
PeV-A3	152037	+	−	+
PeV-A3	K251181-02	+	+	+
PeV-A4	K2511776	+	−	+
PeV-A4	110402	+	−	+
PeV-A5	20552322	+	−	+
PeV-A6	47	+	+	+
PeV-A6	89	+	+	+
CVA9	Griggs	−	−	−
CVB2	Ohio-1	−	−	−
CVB3	Nancy	−	−	−
CVB5	Faulkner	−	−	−
E2	Cornelis	−	−	−
E11	Silva	−	−	−
E20	JV-1	−	−	−
E30	Bastianni	−	−	−

### Specificities of scFv antibodies against PeV types 1–6 and some enteroviruses

**Figure 4:**
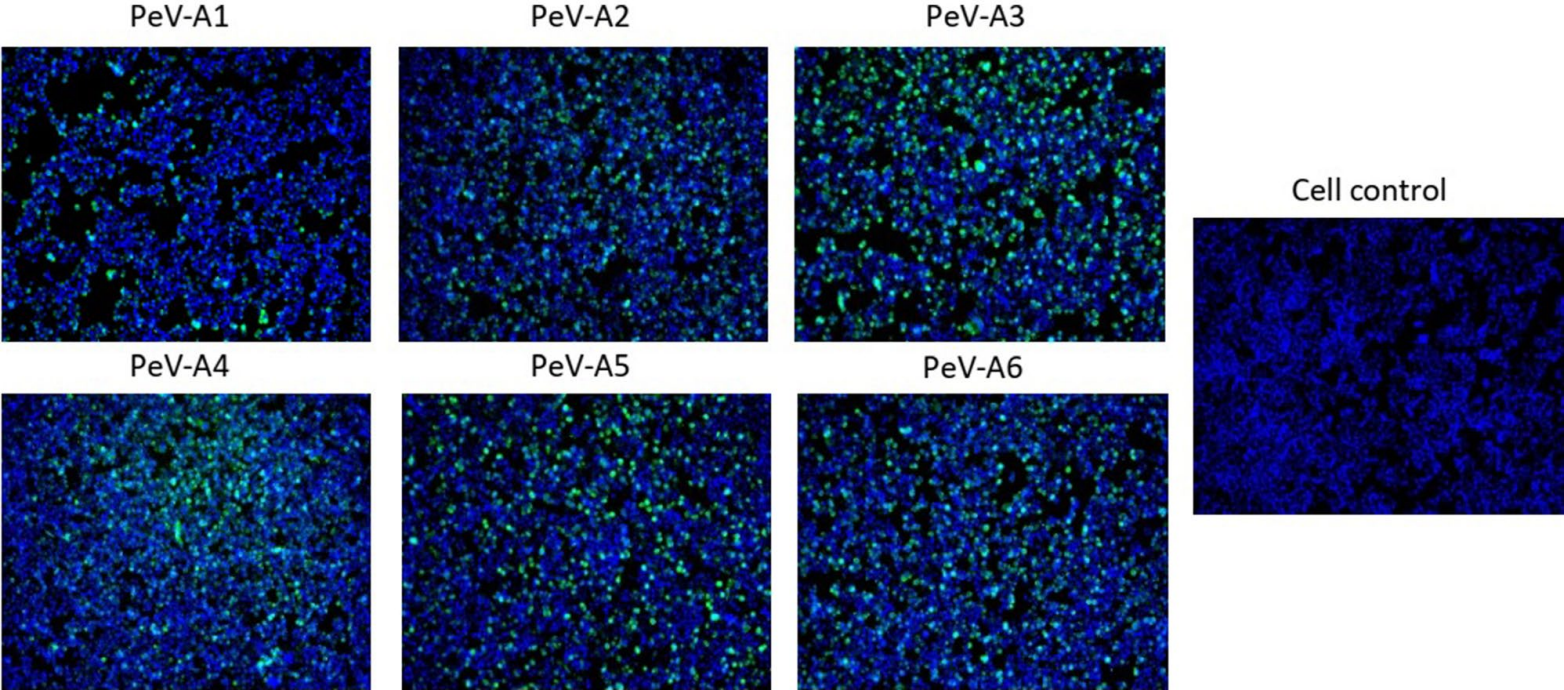
Detection of PeV types 1 to 6 using scFv-71 in IFA. HT-29 cells were mock-infected or infected with PeV types 1 to 6 and stained with scFv-71 antibody followed by staining with rabbit anti-FLAG antibody and anti-rabbit Alexa Fluor 488 secondary antibody. Nuclei were stained with DAPI. Stained cells were visualized using EVOS FL Auto imaging microscope (10 × magnification). Virus strains used in the panel: PeV-A1 “Harris”, PeV-A2 “Williamson”, PeV-A3 “152037”, PeV-A4 “K2511776”, PeV-A5 “20552322” and PeV-A6 “89”.

Single chain Fv antibodies were tested further against PeV-1 to 6 and some enteroviruses using IFA. IFA images are shown for scFv-71 (Fig. 4) and collectively for both scFv-55 and scFv-71 in Table 2. Both scFv antibodies recognized all PeV types. Similar binding profiles between the clones scFv-55 and scFv-71 are not surprising since their amino acid sequences differ only in a total of five positions and both have a short CDRH3 loop of same length (Table 1). In contrast, scFv-59 differs considerably in sequence from the two other clones, and fails to detect one of the PeV-A3 isolates as well as PeV-A4 and PeV-A5 (Table 2).

Cross-reactivities of scFv antibodies to enteroviruses were also tested with IFA. RD cells, which have been shown to be susceptible for enteroviruses^17,18^, were infected with total of eight enterovirus types (CVA9, CVB2, CVB3, CVB5, E2, E11, E20 and E30) and stained with scFv antibodies. Pan-enterovirus antibody (Light Diagnostics, Millipore) was used as a control antibody to specifically detect the enterovirus types. None of the scFv antibodies recognized enterovirus types indicative of their specificity against parechoviruses (data not shown). Specific reactivity was not surprising because parechovirus-specific antibodies were obtained using PeV-A1-VP0 recombinant protein as a target, which is highly different from enteroviral proteins. In general, the identity of the PeV-A1 polypeptides with corresponding proteins of other picornaviruses varies between 14 and 35%^19^, and therefore cross-reactivities were not expected. In all, the data suggest that three different scFv binders have broad range specificity to human parechovirus types.

### Identification of scFv antibody epitope

To identify the epitope for scFv antibodies, a 15-mer peptide library was generated using PeV-A1-VP0 protein as a model^8^. The Pepscan’s CLIPS analysis identified two epitopes. First, a low affinity epitope at location 140-ELPKVFWDHQDK-151. Secondly, a high affinity epitope with a significant signal yield was seen at location 184-LVVYEPKPVVTYDSKLEFGAFT-205, which was narrowed down to a more exact epitope core at position 192-VVTYDSKL-199, based on the CLIPS analysis signal peaks. Structural locations of the high and low affinity epitopes within VP0 can be seen in Supplementary Figure S6. Sequences of 25 PeV isolates representing types 1, and 3–6 (PeV-1 *n* = 20, PeV-3–6 *n* = 1 each) were analysed for the low and high affinity epitopes. Sequencing efforts resulted in 24 assemblies that covered the VP0 region, with PeV-2 (Williamson strain) not resulting in a usable assembly, however, a Williamson strain sequence (AJ005695) was included from GenBank for the analyses including all available PeV VP0 sequence data. Sequence data for the generated assemblies were deposited to GenBank^20^ under accession numbers ON497122–ON497145. All sequenced isolates were identified for a previously described monoclonal antibody P5C4^8^. Sequence analysis of these epitopes from the 24 assemblies found the exact low affinity epitope ELPKVFWDHQDK as indicated by CLIPS analysis in one sequence, PeV-1 prototype Harris, out of 24 total sequences. Pairwise identity of the low affinity epitope was 86.7%. The high affinity epitope core VVTYDSKL was found in 17 out of 24 sequences, with all 17 sequences belonging to PeV-1. Remaining sequences contained V192A, T194D, Y195H, D196S/E, S197D/T, and L199M mutations. Pairwise identity of the high affinity epitope was 76.3% across all sequences. The fact that the high affinity epitope resulted in positive hits for a previously developed antibody for all PeV types 1–6 indicated that the epitope has a degree of flexibility. As such, a deeper sequence and structural analysis of high affinity epitope was done with additional data obtained from GenBank that covered the VP0 region.

### Sequence and structural analysis

**Figure 5:**
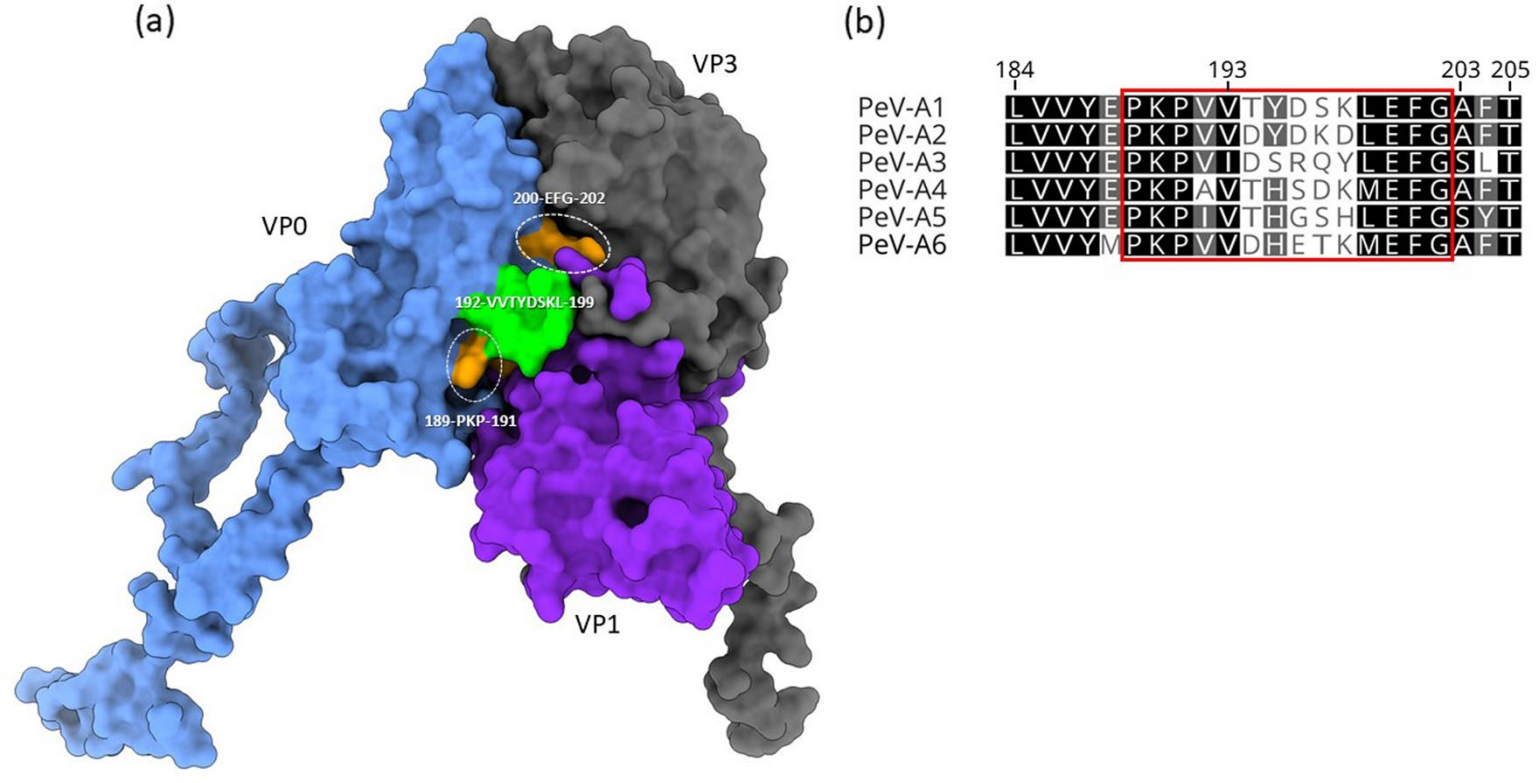
Consensus sequence alignment and location of the epitope on a parechovirus capsid. (**a**) Top-down surface view of PeV-1 structure (PDB: 4z92) asymmetric unit consisting of VP0 (blue), VP1 (magenta), and VP3 (grey) proteins. The core of the putative epitope is colored in green, while the residues of the conserved flanking regions that exhibit surface exposure are colored in orange. Structurally the epitope is located near the junction of the three capsid proteins, which is also structurally close to the RGD motif in VP1, known to be important for integrin binding. The highly conserved flanking regions of the epitope are likely important in stabilizing the base structure of the epitope, which itself has a higher degree of flexibility. (**b**) Sequence alignment of the VP0 residue range obtained from Pepscan’s CLIPS analysis for PeV-1–6 with residues numbered based on the PeV-A1 (Harris) prototype sequence (acc. no. L02971). The area boxed in red represents residues with surface exposure. The flanking regions of the core, VVTYDSKL, are seen highly conserved across different PeV type, while the core alignment itself shows higher flexibility.

Global sequence analysis focusing on the high affinity epitope included all available high quality sequence data covering the VP0 region across all PeV types (*n* = 361) together with the previously assembled sequences (*n* = 24). Structural analysis of the PeV capsid proteins carried out in UCSF ChimeraX v1.2.5^21^ using PeV-A1 crystal structure obtained from the Protein Data Bank^22,23^ (PDB ID: 4z92) as a reference. The high affinity epitope core VVTYDSKL had a pairwise identity of 50% across the full data set (*n* = 385). In total, the exact sequence was found in 107 sequences (PeV-1 *n* = 103, PeV-3 *n* = 1, PeV-8 *n* = 1, PeV-17 *n* = 1, untyped PeV-A *n* = 1). For PeV types 1–6, the pairwise identities for the high affinity epitope were 94.7% for PeV-1 (*n* = 121), 100% for PeV-2 (*n* = 7), 97% for PeV-3 (*n* = 173), 97.7% for PeV-4 (*n* = 22), 82.1% for PeV-5 (*n* = 18), and 83% for PeV-6 (*n* = 21). Evidently, even though the high affinity epitope core exhibits a high degree of variability across all PeV types, the epitope is highly conserved in PeV-1, while also being found in other PeV types. Structural analysis revealed the high affinity epitope to be located towards the middle of the asymmetric subunit, at the junction of VP0, VP1, and VP3 capsid proteins (Fig. 5a). Furthermore, the epitope core exhibits significant surface exposure with 7 out of 8 residues being accessible from the cytoplasmic side of the capsid through the helix and loop structures present. Interestingly, even though the epitope core itself has a high degree of variability across different PeV types, the flanking residues of the region are fully conserved in the full data set (*n* = 385). The fully conserved residue triplet 189-PKP-191 flanks the epitope upstream, and the triplet 200-EFG-202 flanks the epitope downstream. Furthermore, recent bioinformatic prediction analyses of PeV-1 and PeV-3 conformational epitopes by others suggest an epitope involving the VP1 C-terminus, which is also located in the capsid protein junction, and consequently also very close to the high affinity epitope^24^. Moreover, the VP1 C-terminal residues located at the capsid junction, and near the high affinity VVTYDSKL epitope, are only 3 residues upstream from the VP1 RGD motif, which is known to be important for integrin receptor binding in PeV types 1, 2, 4, 5 and 6. Multiple sequence alignment done with Geneious Prime 2021.0.3^25^ against PeV types 1–6 consisting of all available VP0 data shows a higher degree of sequence variation in the epitope core (Fig. 5b). However, the epitope core residues remain very highly conserved within each PeV type itself. In contrast, the flanking regions of the core residues show a high degree of conservation across all PeV types 1–6, even when extending the alignment to the full range of residues with significant signal yield as identified by Pepscan’s CLIPS analysis (Fig. 5b). The conserved regions flanking VVTYDSKL are buried on the very 5’ and 3’ ends of the structure, while the residues triplets mentioned earlier, directly flanking the epitope on both sides, show significant surface exposure together with the VVTYDSKL core region. The fact that these exposed residues also remain fully conserved across all PeV types suggests their importance in potentially stabilizing helical loop structure that forms the high affinity epitope. Furthermore, in this study the epitope was successfully identified with PeV types 1–6 regardless of the inter-type sequence variability seen in the core VVTYDSKL region. This could indicate that the core of the epitope allows for a degree of flexibility on residue level, while the conserved flanking amino acids are important in stabilizing the base structure without affecting its functionality, and perhaps forming a discontinuous, conformational, epitope. As such, based on standard diagnostic assays, this epitope could then be used to detect all PeV-1, and potentially other PeV types 2–6, albeit perhaps with weaker binding affinity.

## Discussion

Parechoviruses (PeVs) are frequent human pathogens, and parechoviral infections are particularly common during the first years of life^26–29^. Although parechoviruses are diagnosed by RT-qPCR, the ongoing covid-19 pandemic has taught us that there is room for other assay types, such as lateral flow assays, in which antibodies play a central role^30^. Therefore, new tools for PeV-A diagnosis are urgently required. However, despite their prevalence and potential as disease agents, there are not many antibodies available for parechoviral studies or diagnosis. PeV-A1-VP0 protein has been shown to be immunogenic^13,31^, and therefore, VP0-derived peptide has been used to develop polyclonal antiserum^14,32^. However, this peptide antibody was only used in detection of a single PeV-A1 isolate. Previously, it has also been reported that rabbit antiserum raised against whole PeV-A1-VP0 (Harris strain) protein possesses diagnostic potential. However, antiserum was tested only against PeV-A1 because only two parechovirus types were known at the time (Harris strain)^13^. Follow-up study demonstrated that this antiserum also binds to cultivable parechovirus types (PeV-A1–A6) suggesting a common epitope within VP0 antigen^8^. The same recombinant antigen was used to generate a monoclonal antibody, Mab P5C4, which was shown to bind to a conserved antibody epitope^8^.

In the current work, we isolated three pan-parechovirus (scFv) antibodies from phage display scFv antibody library against recombinant PeV-A1-VP0 (Harris strain) protein antigen. These antibodies recognized cultivable PeV-A1–A6 prototypes and/or some clinical isolates, and were highly specific to parechoviruses because none of them cross-reacted with related enterovirus types. Furthermore, the study revealed a conserved antibody epitope prevalent in many sequenced parechoviral isolates. Interestingly, this epitope was practically identical to the epitope bound by Mab P5C4^8^. The high affinity epitope was located in the junction of VP0, VP1, and VP3 proteins of the asymmetric subunit. Across all PeV types, the high affinity epitope core has a high degree of sequence flexibility, although the epitope remains well conserved within individual PeV types. The epitope is flanked by fully conserved amino acid triplets on both sides that also have exposure to the capsid surface (Fig. 5b). The fully conserved nature of the flanking region of the epitope suggests that they are important in the function of the epitope, perhaps to structurally stabilize the otherwise variable core region of the epitope. The fact that diagnostic assays resulted in recognition of various PeV types, while we also observed sequence variation in the epitope core itself, could perhaps be explained by the less stringent binding conditions present in the IFA assays, that also better match real biological conditions. This in turn could result in enabling lower affinity binding to other PeV types, regardless of the sequence flexibility observed in the epitope core. As such, the full epitope region could be acting as a conformational epitope, where the conserved flanking regions play an important part.

We conclude to have isolated novel parechoviral antibodies that bind to a putative conformational epitope capable of recognizing all PeV-1 s, with potential to recognize other PeV types 2–6. Despite the sequence variation, we described the usefulness of these antibodies in standard diagnostic assays such as ELISA, and believe that phage display technology paves the rapid way in development of pan-parechovirus antibodies for diagnostics.

## Methods

### Viruses, cells, antibodies and proteins

Parechoviruses were obtained from different sources: PeV-A1 (Harris strain) was from ATCC, “152478”, “452252” and “350757” were gifts from Dr. Katja Wolthers, AMC Amsterdam, “3”, “7”, “8”, “11”, “14”, “19”, “22”, “33”, “35”, “78” were from Dr. Sisko Tauriainen (University of Turku, Finland), and novel PeV isolates “153-20”, “101-17”, “103-2”, “125-7”, “145-12”, “150-8” were isolated from the sample collections of National Institute of Health and Welfare Finland. PeVA-2 (strain Williamson was from ATCC, PeV-A3 “152037”, PeV-A4 “K251176” and PeV-A5 “20552322” were from Dr. Katja Wolthers. PeV-A6 “89” was from Dr. Sisko Tauriainen. Enterovirus prototypes (CVA9, CVB2, CVB3, CVB5, E2, E11, E20 and E30) were originally obtained from ATCC. HT-29 colorectal adenocarcinoma and rhabdomyosarcoma (RD) cell lines were maintained in Dulbecco’s Modified Eagle medium (DMEM) supplemented with gentamicin and 10% Fetal Bovine Serum (FBS). Production and purification of rabbit antiserum generated against PeV-A1 virus particles and PeV-A1-VP0-GST protein has previously been described^8^. Anti-FLAG antibody that recognizes FLAG tag on scFv antibodies was from Cell Signaling Technology (#2368). Light Diagnostics pan-enterovirus reagent (#3360) was from Millipore. Horse radish peroxidase (HRP)-conjugated anti-rabbit antibody was from Jackson Immuno Research (111-035-003) and TMB substrate was from Thermo Fisher (34018). Alexa Fluor 488-labeled anti-rabbit and anti-mouse secondary antibodies (Life Technologies #A-11008 and #A-11001, respectively), as well as DAPI (Sigma Aldrich D1306) were used in immunofluorescence assays and Licor IRDye® 800CW was used in Western blotting.

### Phage display selections for the production of anti-PeV scFv antibodies

PeV-A1-VP0 was biotinylated with Ez-link sulfo-NHS-biotin (Thermo Fisher Scientific) according to manufacturer’s instructions. PeV-A1-VP0 specific binders were selected by phage display from synthetic single-chain antibody fragment (scFv) phage libraries ScFvM^16^ and ScFvP^33^. Both of these libraries use a single human scFv gene as a framework and display the antibody as a fusion to the truncated format of p3 M13 phage coat protein. The libraries contain diversity at the hypervariable CDR regions, but have distinct binding profiles^16^. The methods used for M13 phage display have been previously described^16^. In brief, purified, biotinylated recombinant PeV-A1-VP0 was first immobilized on Dynabeads® M-280 paramagnetic streptavidin beads (Life Technologies). Before the actual panning step where the phage antibody library was incubated with bio-GST-VP0 bound to the M280-beads, negative selections against mere M280-beads and the beads with bio-GST were done to deplete the library from binders against streptavidin and GST. In the panning, 10 µM biotin and 50 µg/ml GST were added as blockers to prevent bead clustering through the biotinylated antigen and enrichment on binders against GST in the panning antigen. At the first selection round 5 × 10^12^ colony-forming units (cfu) of each of the two scFv phage libraries were used as mixed. The mass of antigen-coupled beads used for the first and second round selections was 0.5 mg or 0.05 mg, respectively. The phages were incubated with the beads in TBS (50 mM Tris, 150 mM NaCl, 1% BSA, pH 7.5) containing 1% bovine serum albumin (BSA) for 30–60 min at room temperature with rotation. The unbound phages were removed by washing three times with the same buffer, followed by one wash with TBS + 0.05% Tween-20. Elution of the bound phages was performed with trypsin. Enrichment of specific phages was monitored by a phage immunoassay^16^.

### Construction of vector pLK06FT

The gene cassette in the vector pLK06FT has the following orientation: *Sfi*I (restriction enzyme site)—scFv—*Sfi*I/*Ava*I—AP (bacterial alkaline phosphatase)—TEVPro (protease cleavage site)—His6—Gly-Ser-Gly-linker—FLAG—STOP—*Hind*III. The vector pLK06FT was modified from the previously described vector “His-FLAG-pEB06”^34^ (hereafter called pLK03FT) as follows. The two *Sfi*I cloning sites and bacterial alkaline phosphatase (AP) gene were amplified from vector pAK600^35^ using primers WO375 (5′-TCACACAGGAAACAGCTATGAC-3′) and EB195_ALPrev (5′-ATTGCTCTTCTCTCTTTCAGCCCCAGAGCGGCTTTC-3′) that added *Bsp*QI site to 3’ end after the AP gene. The TEVPro—His6—Gly-Ser-Gly-linker—FLAG—STOP—HindIII containing fragment was PCR amplified from the vector pLK03FT using primer EB914_TEVfor (5′-ATTGCTCTTCAGAGAACCTCTACTTCCAATCGCACC-3′) that added *Bsp*QI site to the 5′ end and primer pAK400 rev (5′-CGCCATTTTTCACTTCACAG-3′). The PCR products were purified, digested with *Bsp*QI, purified again with PCR purification kit (Thermo Fisher Scientific) and joined by T4 DNA ligase (Thermo Fisher Scientific). The ligation product was PCR amplified using primers WO375 and pAK400rev, PCR purified, digested with *Eco*O109I and HindIII, gel extracted and cloned in vector pLK06H^16^.

### Cloning of FLAG-tagged scFv constructs

After two and three rounds of phage display selection, the scFvs genes originating from the human synthetic scFv antibody library were cut off from the phagemid vector (pEB32x/scFv) by SfiI digestion and cloned as a pool into the vector pLK06FT for single-clone immunoactivity screening^16^. Electro-competent *E. coli* XL1-Blue cells (Stratagene) were transformed, and scFv-AP fusion proteins with C-terminal His6 and FLAG tags were expressed in the volume of 200 µl in a 96-well format as described earlier^36^. The immunoreactivities of scFv antibody fragments were determined on streptavidin microtitration plates (Uniogen Oy, Turku, Finland) bound with biotinylated GST-VP0. As a control, binding was also measured to biotinylated GST and streptavidin well only. ScFv-APs were bound from lysed culture supernatants for 1 h. After four washes pNPP substrate was added, plates were shaken 3 h at room temperature and A_405_ was measured. In second screening step binding of eleven IMAC (immobilized metal affinity chromatography) -purified scFv-antibody clones to VP0 protein was confirmed using Eu-labelled anti-AP antibody in TRF assay. The scFv-PeV antibodies were expressed in *E. coli* periplasm in 50 ml shake flask cultures induced o/n at 26 °C with 100 µM IPTG. Cells were harvested and lysed, and scFv was IMAC-purified with HisPur™ Ni-NTA Spin Columns, 0.2 mL resin bed (Thermo Scientific, Espoo, Finland).

### Sandwich ELISA

Corning High binding 96 well plate (#3590) was coated with three scFv-PeV antibodies (scFv-55, -59, and -71) (500 ng/well) in PBS and incubated at 4 °C overnight. The plate was washed three times with PBS and blocked with 3% BSA in PBS for two hours at room temperature followed by washing with PBS. Purified PeV-A1 (Harris strain), control cell lysate, purified VP0-GST fusion protein and purified GST were diluted with PBS and added onto the wells (200 ng/well in PBS). After 90 min incubation at RT, plate was washed three times with PBS, and rabbit anti-PeV-A1 antiserum (pAb-PeV-A1) was added onto wells. Antiserum was incubated 1 h at RT followed by washing with PBS. HRP-conjugated anti-rabbit secondary antibody was added and incubated for 1 h followed by washes with PBS. TMB substrate (Pierce, USA) was added onto the wells and incubated at 37 °C for 30 min which after 0.45 M H_2_SO_4_ was added. OD450 was read with Victor^3^ multilabel counter (PerkinElmer, Turku, Finland).

### Western blot

Lysate of PeV-1 infected HT-29 cells, purified VP0-GST fusion protein, HT-29 cell lysate and purified GST protein were heated in SDS sample buffer at 95 °C for 5 min, and run in 4–20% SDS PAGE (Bio-Rad 4–20% Mini-Protean® TGX™). Chameleon Duo (Licor 928-60000) was used as a marker. Separated proteins were transferred to membranes (Amersham™ Protran™ Nitrocellulose Blotting Membrane, Life Science #10600001) with TransBlot® Semi-Dry Transfer Cell (Bio-Rad). The blotted membranes were blocked overnight with TBS containing 5% BSA followed by blotting with 10 µg/ml of either scFv-55, -59 and -71 antibody in 1% BSA in TBS-T for 1 h. After washes with TBS-T, membranes were incubated with anti-FLAG antibody for 1 h. Membranes were then incubated with anti-rabbit secondary antibody (Licor IRDye® 800CW) for 1 h followed by washes and analysing with Odyssey (Licor). Full membranes were blotted and the area where all marker bands were visible was cropped together with the target band. Thus, any background binding is evident in the image. Experiments were repeated once.

### Immunofluorescence assay

In infection assay, DMEM was supplemented with 1% FBS. Cells were seeded at 10 000 per well and grown on black 96-well plates (6005182, Perkin Elmer) to 90% confluency. The cells were infected with a virus dilution aiming at 20–40% efficiency of infection, which make FFUs (fluorescence-forming units). The infection was continued for 6 h at 37 °C and 5% CO_2_ followed by fixing with 4% formalin and permeabilization with 0.2% Triton X-100. Infected, fixed and permeabilized cells in 96-well plates were stained as follows: scFv-55, -59 and -71 antibodies were diluted to 3% BSA in PBS to concentration of 1 µg/ml and incubated on the cells for 1 h. After washes with PBS, anti-FLAG antibody was added and incubated for 1 h followed by washes with PBS. Alexa Fluor 488-labeled anti-rabbit secondary antibody was incubated for 1 h. With enterovirus testing pan-Enterovirus antibody was used as a primary antibody and Alexa Fluor 488-labeled anti-mouse was used as a secondary antibody. The nuclei were visualized with DAPI. 96-well plates were visualized by fluorescence microscope using EVOS FL Auto with 10 × objective (Thermo Fisher Scientific), and the brightness and contrast were adjusted with EVOS FL and Gimp 2.0. softwares.

### Epitope mapping

Epitope mapping was performed at Pepscan Presto BV (Lelystad, The Netherlands) using Pepscan’s proprietary Chemically Linked Peptides on Scaffolds (CLIPS) technology. 15-mer peptides from the VP0 protein (corresponding to nucleotides 710-1576 of PeV-A1 Harris strain; GenBank acc. no. L02971) were generated with an offset of one amino acid. scFv antibodies were incubated with the peptides immobilized on a glass slide (peptide library) in 4% horse serum, 5% ovalbumin (w/v) in PBS/1% Tween. After washing, the peptide library was incubated with a 1/1000 dilution of anti-FLAG antibody conjugate for one hour at 25 °C, washed again, and incubated with the peroxidase substrate 2,2′-azino-di-3-ethylbenzthiazoline sulfonate (ABTS) and 2 μl of 3% H_2_O_2_. After one hour, the color development was measured and quantified with a charge coupled device (CCD) camera and an image processing system^37^.

### Sequence and structural analyses

All non-redundant human parechovirus sequences covering the VP0 region were retrieved from GenBank (*n* = 361) and combined with a sequenced set of parechoviruses of types 1 and 3–6 (*n* = 24) from this study. Multiple sequence alignments were carried out with Geneious Prime 2021.0.3^25^ using the MUSCLE^38^ [4] plugin with default settings. Structural analysis of the epitope was carried out using UCSF ChimeraX v1.2.5^21^. The structure of human parechovirus 1 virion obtained from the Protein Data Bank (PDB ID: 4z92)^22,23^ was used as a reference to determine the exact location and surface exposure of the epitope.

## Supplementary Information

Supplementary Information.

## Data Availability

The sequence data generated during the current study are available in the GenBank repository (https://www.ncbi.nlm.nih.gov/genbank/) through accession numbers ON497122–ON497145. Original Western blot images for the current study are available in the supplementary information files.
