# Supplementary material for: Isolation and characterization of phage display-derived scFv antibodies against human parechovirus 1 VP0 protein
Source: Sci Rep. 2022 Aug 4;12:13453. doi: 10.1038/s41598-022-17678-y (PMC9352675; doi:10.1038/s41598-022-17678-y)
Supplement: Supplementary file 1 — Supplementary Information. [file 41598_2022_17678_MOESM1_ESM.pdf]

Supplementary information for

Isolation and characterization of phage display-derived scFv antibodies against human parechovirus 1 VP0 protein

Eero Hietanen[1,3], Lav Tripathi[1,3], Eeva-Christine Brockmann[2], Pirjo Merilahti[1,#a], Urpo Lamminmäki[2] and Petri Susi[1,*]

[1]Institute of Biomedicine, University of Turku, Turku, Finland

[2]Department of Biotechnology, University of Turku, Turku, Finland

[3]These authors contributed equally: Eero Hietanen and Lav Tripathi

[*]Corresponding author

[#a]Current address: Biovian Ltd, Turku, Finland

Corresponding author:

Petri Susi

Email: pesusi@utu.fi

Tel: +358 29 450 4556

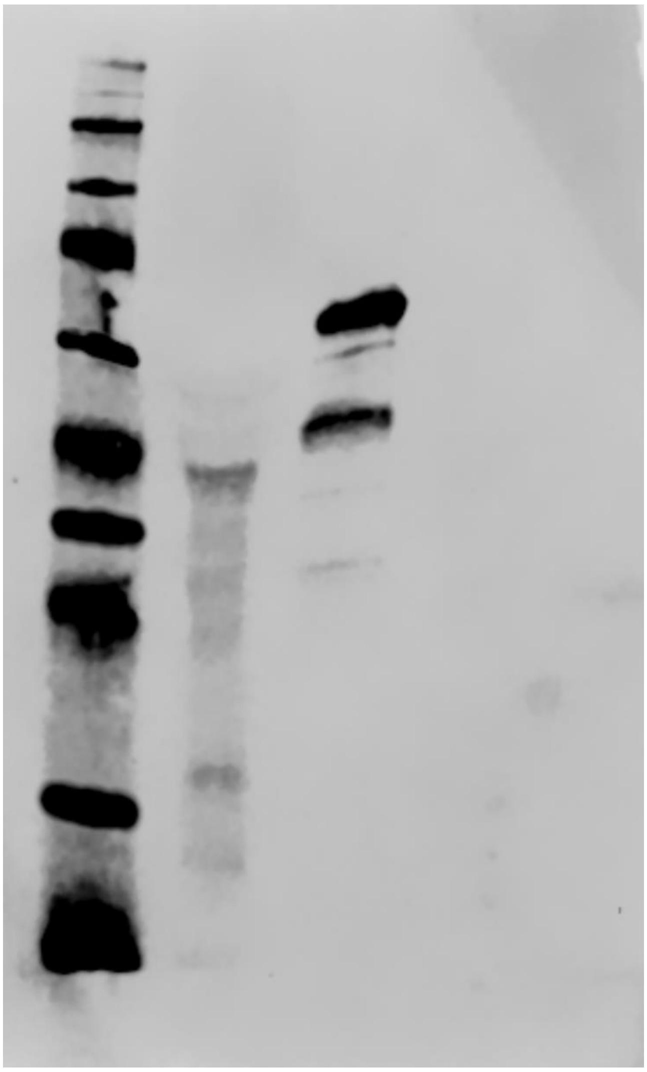

Figure S1. Original Western blot image for scFv-55. Lanes from left to right are: marker; lane 1: PeV-A1 (Harris strain); lane 2: VP0-GST; lane 3: Cell lysate; lane 4: GST.

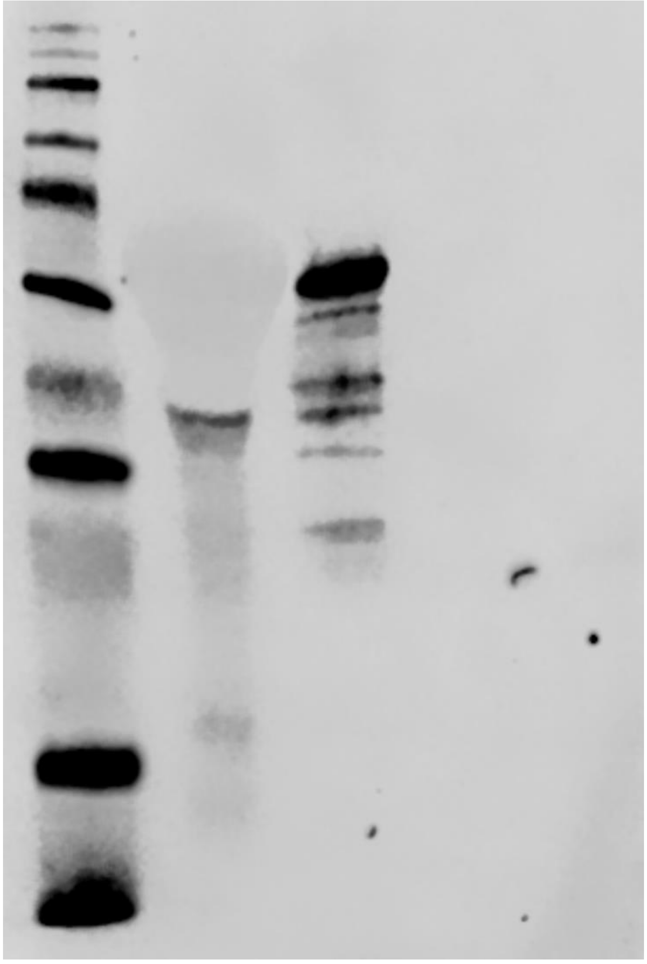

Figure S2. Original Western blot image for scFv-59. Lanes from left to right are: marker; lane 1: PeV-A1 (Harris strain); lane 2: VP0-GST; lane 3: Cell lysate; lane 4: GST.

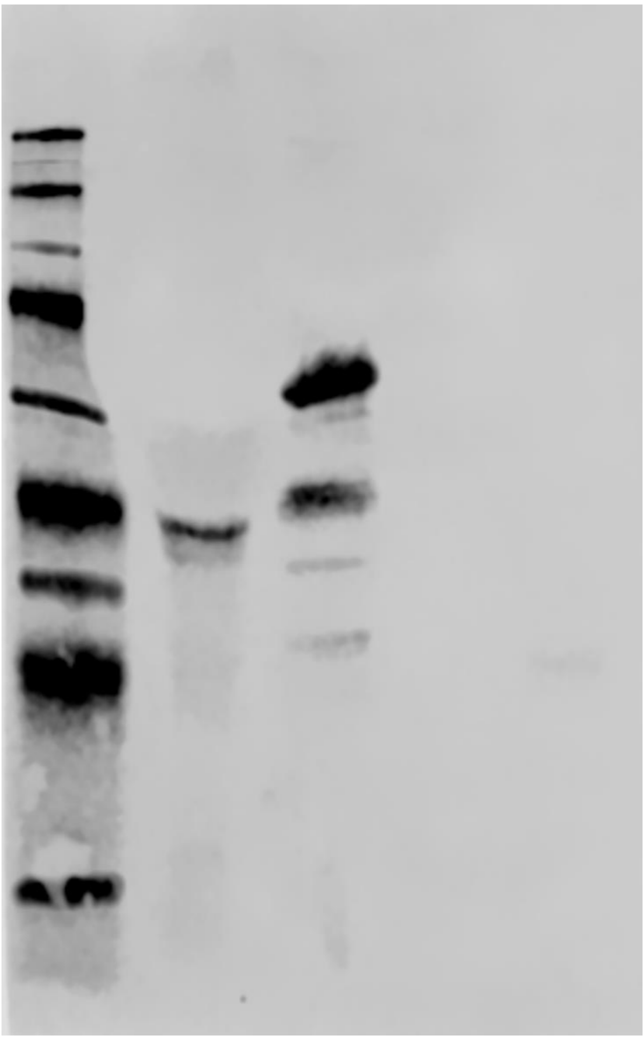

Figure S3. Original Western blot image for scFv-71. Lanes from left to right are: marker; lane 1: PeV-A1 (Harris strain); lane 2: VP0-GST; lane 3: Cell lysate; lane 4: GST.

ANTIBODIES SCFV C-55, SCFV-59, AND SCFV C-71

When tested under high stringency conditions ScFv C-55, ScFv C-59 and ScFv C-71 similarly bound linear overlapping peptides with core sequence $_{191}$PVVTYDSKL$_{198}$ (Figure 7). Additional peaks of lower intensity were observed for all three antibodies. One of the major peaks was attributed to linear peptides with core sequence $_{140}$ELPKVFWDHQDK$_{151}$, which shares three conserved and two semi-conserved residues with the dominantly recognized sequence (ClustalW alignment is shown below).

```
ELPKVFWDHQDK
--PVVTYDSKL-
  *  *  :*  :
```

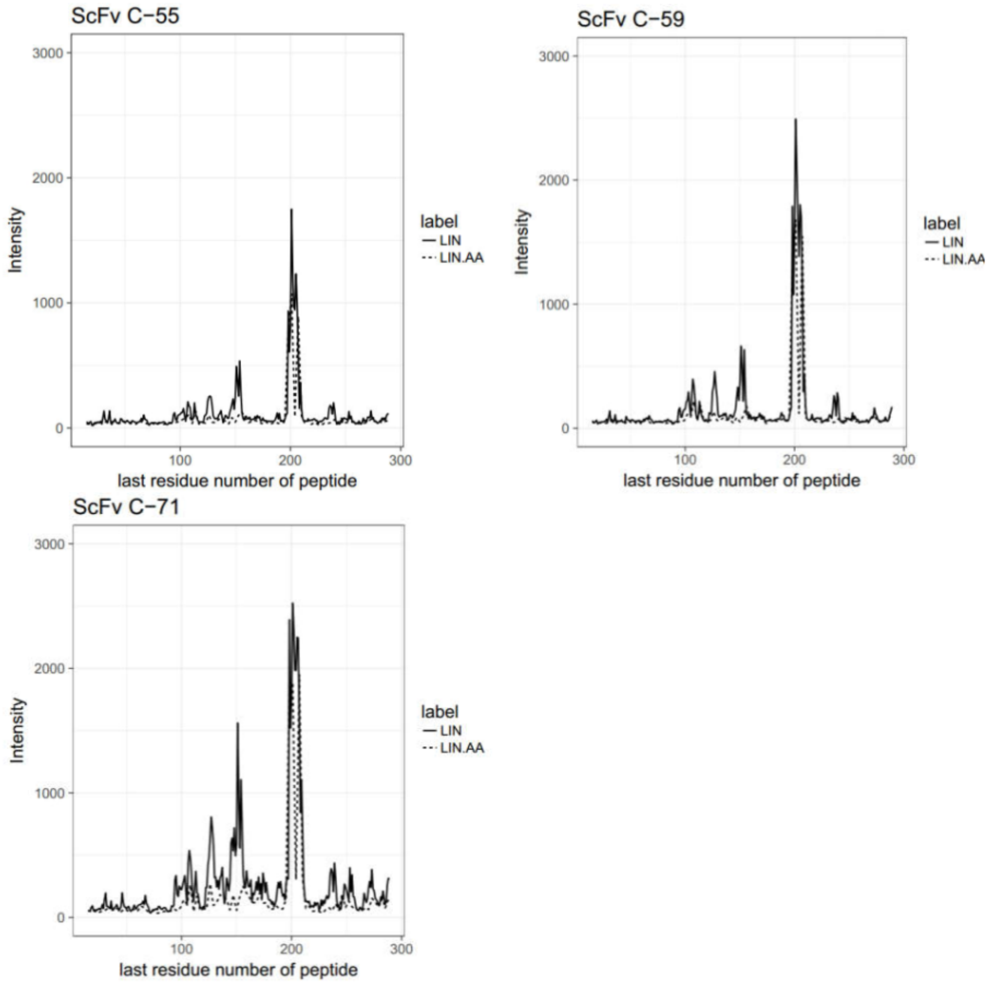

Figure S4. Intensity profiles recorded by the Pepscan's CLIPS analysis as taken from the original report.

```
                                          * *
          VL-FR1                   CDR1       VL-FR2      CDR2 VL-FR3
scFv-55  EIVLTQSPGTLSLSPGERATLSCRASQSVSSSYLHWYQQKPGQAPRLLIYGASSRATGVPDRFSG
scFv-59  ----------------------------------N------------------------------
scFv-71  -----------------------------------------------------------------

                                 * **** *
          VL-FR3                CDR3      VL-FR4         G4S-linker
scFv-55  SGSGTDFTLTISRLEPEDFAVYYCLQDNYDPPTFGQGTKVEIKRTGGGGSGAGGSGGGGTGGGGS
scFv-59  ------------------------Q-TY-I-L---------------------------------
scFv-71  ----------------------------SA-L---------------------------------

                                       * * *              * **** * *
          VH-FR1                   CDR1       VH-FR2        CDR2    VH-FR3
scFv-55  EVQLLESGGGLVQPGGSLRLSCAASGFTFSSYGMDWVRQAPGKGLEWVSRITPSGGSTYYADSVK
scFv-59  --------------------------------A-S--------------S------EHG------
scFv-71  -----------------------------------------------------------------

             ¤                          *****
               VH-FR3                  CDR3 VH-FR4
scFv-55  GRFTFSRDNSKNTLYLQMNSLRAEDTAVYYCASGAYWGQGTLVTVSS
scFv-59  ----I--------------------------TGW-------------
scFv-71  ----I-----------------------------YI-----------
```

Figure S5. Single clone sequences. The scFv antibodies were isolated from antibody library composed of a single framework with human KV3-20 and HV3-23 joined with a 20 aa Gly-Ser linker. The structure of the library has been previously described by Huovinen et al.[1]. Full amino acid sequence of clone scFv-55 is shown. In other clones only the residues differing from the scFv-55 are shown. CDR regions are according to IMGT definition[2]. Sites containing sequence variation in the libraries are indicated with (*). An extra sporadic mutation was found in the VH framework outside the randomized CDR regions (¤).

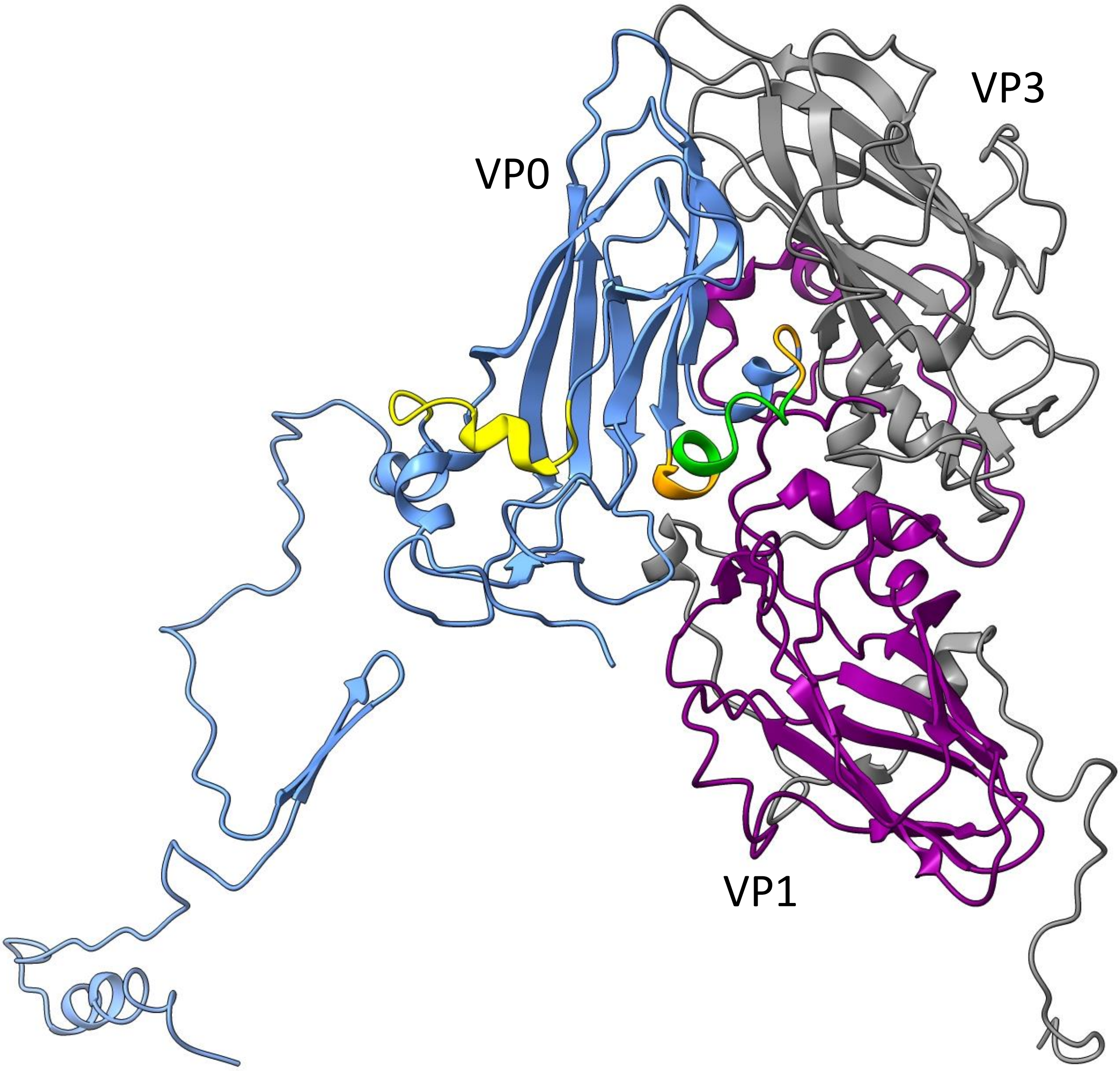

Figure S6. Locations of the high and low affinity epitopes. Top-down ribbon view of the asymmetric unit consisting of VP0 (blue), VP1 (magenta), and VP3 (grey). The high affinity epitope 192-VVTYDSKL-199 (green) is flanked by the fully conserved residue triplets 189-PKP-191 and 200-EFG-202 (orange). The low affinity epitope 140- ELPKVFWDHQDK-151 (yellow) is located further upstream in VP0. Distance between the closest atoms between the high and low affinity epitopes is 12.7 Å.

References

1. Huovinen, T. *et al.* Two ScFv antibody libraries derived from identical VL-VH framework with different binding site designs display distinct binding profiles. *Protein Eng. Des. Sel.* **26**, 683–693. https://doi.org/10.1093/protein/gzt037 (2013).

2. Lefranc, M.-P. Unique database numberings system for immunogenetic analysis. *Immunol. Today* **18**, 509. https://doi.org/10.1016/S0167-5699(97)01163-8 (1997).
